# Molecular Characterization and Antifungal Profiling of Nine Phenotypic *Aspergillus nidulans* Isolates: A Case Series From North India

**DOI:** 10.1002/mbo3.70338

**Published:** 2026-06-21

**Authors:** Aishwarya Nikhil, Atul Kumar Tiwari, Pearl Parashar, Mohit Bhatia, Ragini Tilak, Deepak Kumar, Sudhir Kumar Singh, Roger J. Narayan, Munesh K. Gupta

**Affiliations:** ^1^ Mycology Research Group, Department of Microbiology, Institute of Medical Sciences Banaras Hindu University Varanasi Uttar Pradesh India; ^2^ School of Chemistry Tel Aviv University Tel Aviv Yafo Israel; ^3^ Department of TB and Respiratory Medicine, Sir Sunderlal Hospital (BHU) Varanasi Uttar Pradesh India; ^4^ Viral Research Diagnostic Laboratory, Department of Microbiology, Institute of Medical Science Banaras Hindu University Varanasi Uttar Pradesh India; ^5^ Joint Department of Biomedical Engineering North Carolina State University Raleigh North Carolina USA

**Keywords:** *Aspergillus nidulans*, *Aspergillus stellatus*, Chronic pulmonary aspergillosis, cryptic species, ITS1‐5.8SrDNA

## Abstract

*Aspergillus nidulans* is a fungal pathogen that causes respiratory issues in individuals with compromised immune systems. It is identified through culture characteristics and microscopic features such as Cleistothecia and Hülle Cells. However, similar traits are found in cryptic *Aspergillus* species, such as *stellatus, cristatus*, and *oryzae*. In a case series from a hospital in North India, *A. nidulans* strains isolated from patients with pulmonary aspergillosis were phenotypically identified and then underwent molecular characterization through sequencing of the amplified ITS1‐5.8S rDNA‐ITS2 region and antifungal susceptibility testing (AST) according to the CLSI M38A3 guidelines. The molecular characteristics and antifungal profiles of the nine phenotypic *A. nidulans* were as follows: *A. stellatus* (*n* = 5), *A. nidulans* (*n* = 2), *A. cristatus* (*n* = 1), and *A. oryzae* (*n* = 1). Phylogenetic analysis revealed a close relationship between *A. stellatus, A. nidulans*, and *A. cristatus*, while *A. oryzae* showed significant divergence. Furthermore, the minimum inhibitory concentration (MIC) of antifungals was the lowest for caspofungin, followed by voriconazole. However, a higher amphotericin B MIC (2 µg/mL) was observed for *A. stellatus*. Thus, sequencing the ITS1‐5.8S rDNA‐ITS2 region can accurately identify cryptic species with superior taxonomic resolution. Additionally, the MIC of amphotericin B against *A. stellatus* underscores the importance of precise molecular identification and antifungal MIC profiling in each case of pulmonary aspergillosis.

## Graphical abstract

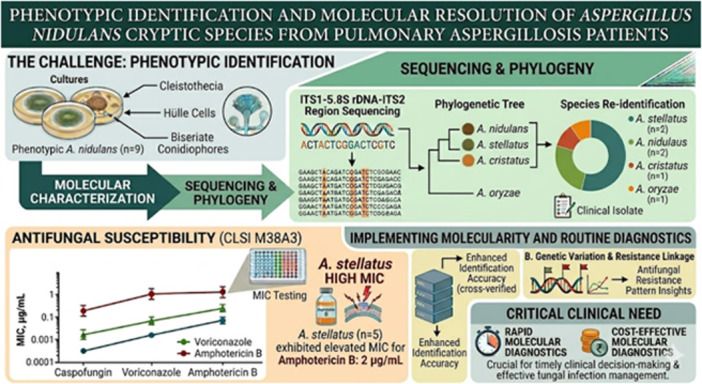

## 1 Introduction

Respiratory aspergillosis is a major contributor to illness and death in countries where TB is widespread, such as India. This condition is divided into allergic bronchopulmonary aspergillosis, chronic pulmonary aspergillosis (CPA), and invasive pulmonary aspergillosis (IPA). CPA primarily affects individuals with existing lung cavities, whereas invasive pulmonary aspergillosis is observed in individuals with weakened immune systems, particularly neutropenia, or those undergoing immunosuppressive treatment for cancer and autoimmune diseases. The fungus responsible for this condition, *Aspergillus*, is an environmental saprotroph whose spores can enter the human body and lead to infections. The emergence of these cryptic species poses significant diagnostic and treatment challenges, as they are difficult to accurately identify using phenotypic methods and often have higher antifungal minimum inhibitory concentrations than their non‐cryptic counterparts. Various *Aspergillus* species, including *fumigatus, flavus, niger, terreus*, and *versicolor*, have been identified as causes of pulmonary aspergillosis, with *A. fumigatus* and *A. flavus* being the most common species (Khan et al. 2024). Additionally, *A. nidulans*, a rare opportunistic fungus, has been linked to pulmonary aspergillosis, particularly in patients with chronic granulomatous disease (CGD) (Pinheiro et al. 2023). Identification of the causative fungus is typically done through phenotypic methods, examining culture characteristics on Sabouraud dextrose agar (SDA) and Czapek dox agar (CDA), and microscopic features such as conidiophores, vesicles, phialides, conidia, cleistothecia, and huelle cells in a lactophenol cotton blue wet mount (LPCB). However, these phenotypic methods are time‐consuming and subject to variations. *A. nidulans* strains are further identified by sequencing targeted DNA regions, such as the ITS, β‐tubulin, or calmodulin genes. Sklenář et al. reported six new *Aspergillus* subgenus Nidulantes using the ITS and large subunit rDNA regions (Sklenář et al. 2020). Similarly, Sun et al. identified four new Chinese *Aspergillus* subgenus nidulantes species through partial sequencing of calmodulin, β‐tubulin, and RNA polymerase II (RNA pol II) genes (Sun et al. 2022). The transition in clinical practice from empirical methods to precision medicine has enhanced the role of diagnostic testing as an important pathway for selecting an effective antifungal therapy. In this context, pulmonary aspergillosis is linked to high mortality rates (between 45% and over 90%), particularly in individuals with compromised immune systems and those in intensive care units, for whom any delay in treatment can have detrimental consequences (Huang et al. 2026). Recently, Mohamadina et al. identified *A. terreus* species as the most commonly detected species, followed by *A. nidulans, A. latus, A. ochraceus,* and *A. citrinoterreus* (Mohamadnia et al. 2020). Examination of the benA gene indicated 12 unique genotypes among the *A. terreus* isolates; in contrast, no intraspecies variation was observed in the other species. CFG showed the lowest MEC_50_/MIC_50_ value of 0.007 μg/mL, followed by POS at 0.125 μg/mL; VRC, ITC, and ISA at 0.25 μg/mL; RAV at 0.5 μg/mL; and AMB at 8 μg/mL. Only 15.5% (7 out of 45) of the isolates were noted to be susceptible to AMB. Therefore, this study involved molecular characterization and antifungal susceptibility profiling on nine phenotypic *A. nidulans* strains that were isolated from patients with pulmonary aspergillosis in order to expand the available data on this clinically relevant species.

## 2 Materials and Methods

### 2.1 Materials

All reagents, antifungal agents, and media used were of analytical grade. Itraconazole (ITZ), amphotericin B (AMB), voriconazole (VOR), ravuconazole (RUV), posaconazole (PCZ), and caspofungin (CAS) were obtained from Sigma‐Aldrich Private Limited (St. Louis, MO, USA). RPMI‐1640 MOPS (3‐(N‐morpholino) propanesulfonic acid) without sodium bicarbonate, Sabouraud dextrose agar (SDA), brain‐heart infusion agar, potato dextrose agar (PDA), Czapek Dox Agar (CDA), ethanol, chloroform, phenol, and iso‐amyl alcohol, were acquired from Hi‐Media (Mumbai, Maharashtra, India). Similarly, sterile plasticware, 96‐well flat‐bottom microtiter plates, centrifuge tubes, and microcentrifuge tubes were purchased from Tarson Products Pvt. Ltd. (Kolkata, West Bengal, India). For the extraction of genomic fungal DNA, the QIAquick gel extraction kit was purchased from QIAGEN (New Delhi, India).

### 2.2 Conventional Diagnosis and Antifungal Susceptibility Profile

The Ethics Committee of the Institute of Medical Sciences approved this study (Dean/2021/EC/3003, October 29, 2021). In our study, we isolated nine phenotypically identified strains of *A. nidulans* from bronchoalveolar lavage samples of patients with respiratory aspergillosis between 2022 and 2024. We gathered demographic information, clinical symptoms, and radiological findings. For mycological analysis, we briefly collected BAL or early morning sputum samples, which were examined for characteristic hyphae using a KOH wet mount and simultaneously inoculated onto blood agar, SDA, and CDA media. The inoculated media were then incubated at 28°C in a BOD incubator. We identified the cultivated fungi using phenotypic methods, assessing colony morphology (growth rate, colony diameter, aerial mycelium, and pigmentation) on CDA, and microscopic features (conidiophore vesicles, cleistothecia, Hülle cells, metulae, phialides, and conidia) using a LPCB wet mount. All isolates were preserved at −20°C in a solution of 15% glycerol and 0.85% normal saline for future studies.

The MIC of AMB, ITZ, RUV, VRC, POS, and CAS were determined against phenotypically identified *A. nidulans* isolates using the broth microdilution method in accordance with the CLSI M38‐A3 guidelines 2008 (CLSI 2008). In summary, the antifungal MIC was determined by introducing 100 µL of 10^4^ conidia/mL into each well of a 96‐well flat‐bottom microtiter plate containing 100 µL of RPMI medium and 100 µL of serially diluted antifungal agents. For quality control, we used *C. krusei* (ATCC 6258) and *C. parapsilosis* (ATCC 22019) (Pa 2002). The MIC for azoles and AMB was defined as the lowest drug concentration that inhibited visible growth, whereas for CAS, it was the MEC that resulted in a compact fungal hyphal structure observable under a brightfield microscope at 400× magnification.

For molecular characterization, we initially extracted DNA from phenotypic *A. nidulans* strains. The mycelial mat was harvested from *A. nidulans*‐like growth on PDA medium, ground in liquid nitrogen using a mortar and pestle, and air‐dried. DNA extraction was performed using the standard phenol: chloroform method. We assessed the quality of the extracted DNA using Nanodrop to determine the A260/280 ratio. Subsequently, we amplified the extracted DNA using panfungal ITS–1 (5′‐TCCGTAGGTGAACCTGCGG‐3′) and ITS–4 (5′‐TCCTCCGCTTATTGATATGC‐3′) primers. The amplification reaction was conducted in a 25 µL volume containing 5 µL of template and 0.25 µL of each primer, with initial denaturation at 95°C for 5 min, followed by 40 cycles at 94°C (1 min), 55°C (30 s), and 72°C (1 min), and a final extension at 72°C for 7 min. A 2% agarose gel electrophoresis revealed an amplicon of approximately 500–600 bp. The PCR product was then eluted using a QIAGEN gel elution kit according to the manufacturer's instructions. For sequencing, 5 µL of the PCR amplicon was treated with Exo SAP solution according to the Applied Biosystem sequencing kit instructions. The amplified product was initially mixed with precipitation reagents (MM1 and MM2), chilled on ice, and centrifuged to form a DNA‐pellet. The pellet was washed twice with ethanol, air‐dried at 37°C, resuspended in Hi‐Di formamide, denatured at 95°C for 5 min, and loaded onto an Applied Biosystems 3500 Genetic Analyzer for sequencing. FinchTV version 1.4.0 was used to generate chromatograms displaying peaks of various colors (Supporting Information S1). We also constructed a phylogenetic tree using MEGA 12 version 12.0.11. For the sequencing analysis, we retrieved the *A. nidulans* strain (NR_133684.1) from NCBI database and used to determine the genetic relatedness among isolates (Camacho et al. 2009).

## 3 Results

During the study period, we isolated 506 unique strains of *Aspergillus* species from patients diagnosed with pulmonary aspergillosis. Among these, only nine strains were phenotypically identified as *A. nidulans* (9/506, 1.77%). Of these nine patients, six were women. The average age of the patients was 34.41 years. Table 1 provides a summary of the patients' demographic information. The identification of these strains was based on their cultural characteristics: moderate to rapid growth of white to dark green velvety colonies on the obverse view of PDA medium, which turned brownish‐green as they aged, and yellowish‐brown growth on the reverse view. On CDA, *A. nidulans* strains exhibited velvety, sometimes floccose colony surfaces with dense, flat, or slightly wrinkled growth patterns and white to cream or occasionally greyish mycelium underneath. In lactophenol cotton blue wet mounts, dull yellow to buff cleistothecia with clusters of Hülle cells and green‐to‐dark‐green conidia were observed. The culture characteristics of the isolates and antifungal susceptibility results are presented in Table 2.

**Table 1:** Clinico‐epidemiological data of the enrolled patients at Sir Sunderlal Hospital, Banaras Hindu University, Uttar Pradesh, India.

S.N.	Age (years)	Gender (M/F)	Specimen	Predisposing risk factor	Clinical manifestation	Clinical diagnosis	Phenotypic identification	Reclassified isolate as per molecular identification
1	36	F	BAL	Bronchial asthma, ABPA, type 2 diabetes mellitus, allergic rhinitis	Productive cough, dyspnea, fever	Chronic pulmonary aspergillosis	*A. nidulans*	*A. stellatus*
2	44	M	BAL	History of inhaler use, asthma	Cough productive sputum, dyspnea,	Chronic pulmonary aspergillosis	*A. nidulans*	*A. stellatus*
3	46	F	BAL	Hypertension	Dry cough, hemoptysis, weight loss	Chronic pulmonary aspergillosis	*A. nidulans*	*A. stellatus*
4	45	F	BAL	Type 2 diabetes mellitus, ATT intake, TB	Fever, chest pain, cough, dyspnea	Chronic pulmonary aspergillosis	*A. nidulans*	*A. stellatus*
5	38	M	Sputum	Addiction history, TB	Chest pain, fever	Chronic pulmonary aspergillosis	*A. nidulans*	*A. stellatus*
6	34	M	Sputum	Hypertension	Hemoptysis, fever, dyspnea	Chronic pulmonary aspergillosis	*A. nidulans*	*A. nidulans*
7	25	F	Sputum	TB	Productive cough, chest pain, fever, dyspnea	Chronic pulmonary aspergillosis	*A. nidulans*	*A. nidulans*
8	35	F	BAL	Type 2 diabetes mellitus, COPD, and biomass exposure	Chest pain, dyspnea, headache, productive cough	Invasive pulmonary aspergillosis	*A. nidulans*	*A. cristatus*
9	22	M	Sputum	SARS‐CoV‐2 infection	Dry cough, chest pain, fever	Invasive pulmonary aspergillosis	*A. nidulans*	*A. oryzae*

**Table 2:** Phenotypic characteristics over Czapek dox medium, molecular, and antifungal MIC data against nine *Aspergillus* strains isolated from lower respiratory tract samples.

Lab no.	Morphology		Microscopy	Gene ID	Molecular identification	AST (MIC/MEC, µg/mL)
	Obverse view	Reverse view	LPCB wet mount 400×			
1001	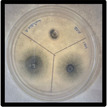 Dark green velvety colonies, spreading outside, yellow spot in between the dark green coloration.	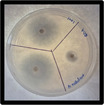 White to cream reverse beneath the colonies	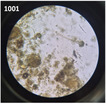 Globular vesicle, biseriate phialide and cleistothecia	PX091582	*A. stellatus*	AMB‐2 ITZ‐0.5 VOR‐1 RUV‐1 PCZ‐0.25 CAS‐0.0625
1124	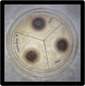 Dark, green‐colored colonies with some green granules scattered throughout.	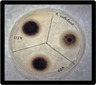 Dark yellow to brown color pigmentation	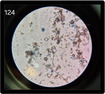 Hülle cells, cleistothecia,	PX248706	*A. stellatus*	AMB‐0.5 ITZ‐0.031 VOR‐0.5 RUV‐0.125 PCZ‐0.125 CAS‐0.125
2714	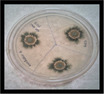 Dark green velvety colonies, spreading outside, yellow spot in between the yellow‐green coloration	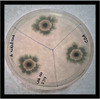 Light yellowish green pigmentation	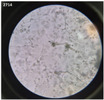 Globular‐shaped vesicle, with biseriate.	PX248709	*A. stellatus*	AMB‐1 ITZ‐0.5 VOR‐0.125 RUV‐0.0625 PCZ‐0.031 CAS‐0.0625
3247	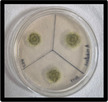 Dark green, dry, powdery colonies with yellow, granular spots all over them.	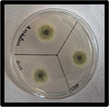 Greenish‐yellow color pigmentation	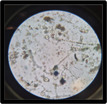 Globular vesicle biseriate phialides, with the presence of Hülle cells.	PX248716	*A. stellatus*	AMB‐2 ITZ‐0.125 VOR‐0.0625 RUV‐1 PCZ‐0.0625 CAS‐0.25
1513	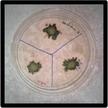 Dark green colonies with yellow granular spots all over them. Dry powdery colonies.	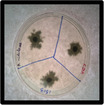 Dark yellow‐green pigmentation	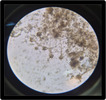 Globular‐shaped vesicle, with conidia, biseriate phialide, and cleistothecia were observed.	PX248713	*A. stellatus*	AMB‐1 ITZ‐0.0625 VOR‐0.5 RUV‐0.031 PCZ‐0.125 CAS‐0.031
1314	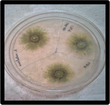 Dry, light green‐yellow colonies with a yellow, granular spot.	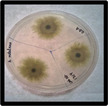 Light yellowish green pigmentation	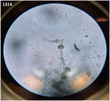 Globule‐shaped vesicle, with biseriate phialides, round conidia, and Hülle cells.	PX248708	*A. nidulans*	AMB‐0.125 ITZ‐1 VOR‐1 RUV‐0.0625 PCZ‐0.0625 CAS‐0.0625
4415	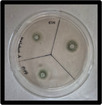 Slow‐growing colonies, characterized by a dark green powder texture, spreading outward, with yellow granules.	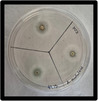 White‐yellow pigmentation.	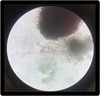 Hyaline smooth conidiophore, globular vesicle, biseriate phialide, and cluster of Hülle cells.	PX248715	*A. nidulans*	AMB‐2 ITZ‐1 VOR‐1 RUV‐0.125 PCZ‐0.125 CAS‐0.125
887	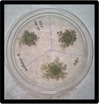 Dry green velvety colonies with a yellow spot due to cleistothecia production	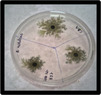 Light yellow to dark yellowish green pigmentation.	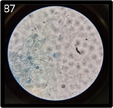 Hyaline smooth conidiophore, globule vesicle, biseriate phialide, and small conidia	PX093629	*A. cristatus*	AMB‐0.5 ITZ‐0.5 VOR‐0.5 RUV‐0.0625 PCZ‐0.25 CAS‐0.031
3475	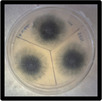 Dark green velvety powdery colonies spreading outside, with small yellow granular spots.	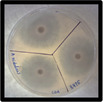 Greyish white pigmentation from the center of the colony to the outside	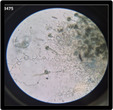 Globular vesicle, biseriate phialide, small, uniform conidia with Hülle cells.	PX363094	*A. oryzae*	AMB‐1 ITZ‐0.125 VOR‐0.125 RUV‐0.031 PCZ‐0.125 CAS‐0.0625

**Figure 1:**
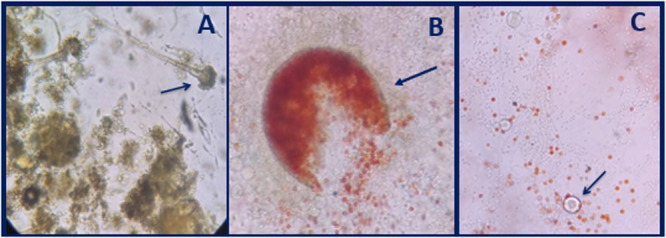
LPCB wet mount of *Aspergillus* isolates reveals: (A) conidiophore having vesicle with biseriate phialides with chain of conidia (arrow), (B) cleistothecia (arrow), and (C) Hülle cells (arrow).

At a magnification of 400× using a lactophenol cotton blue wet mount, we identified septate, branched, and transparent hyphae featuring short stalks known as conidiophores, which had round or dome‐shaped structures called vesicles at their ends. These vesicles were either partially or completely covered with two layers of small cells, metulae, and philides, which generated chains of round, greenish conidia. Additionally, Hülle cells, which are thick‐walled and round, were observed surrounding larger round structures called cleistothecia in all isolated strains (Figure 1).

**Figure 2:**
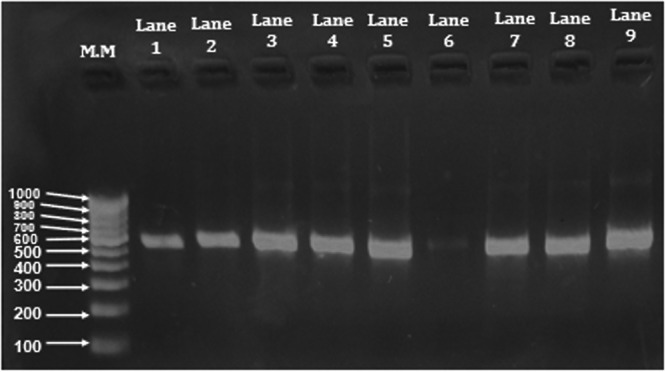
Agarose gel electrophoresis of the ITS PCR products of different *Aspergillus* isolates: lane 1 to 9; 1. *A. cristatus* (AC1), 2. *A. stellatus* (AS1), 3. *A. stellatus* (AS2), 4. *A. nidulans* (AN1), 5. *A. stellatus* (AS3), 6. *A. stellatus* (AS5), 7. *A. oryzae* (AO1), 8. *A. nidulans* (AN2), 9. *A. stellatus* (AS4). Lane MM was a 100 bp molecular size marker.

We conducted molecular identification of these nine *A. nidulans* strains, which were initially identified based on their phenotypic characteristics. To begin, we amplified the conserved ribosomal ITS regions, specifically the ITS1‐5.8‐ITS2 region, resulting in an amplicon of approximately 550 base pairs, as shown in Figure 2. Following this, we used BLAST to identify all the amplified sequences, ensuring a match reliability with a percentage identity greater than 99% and query coverage exceeding 95%, along with the lowest e‐value (Samson et al. 2014). The sequenced data were submitted to the NCBI GenBank (Supporting Information S1: Table S1). In this analysis, AS1, AS2, AS3, AS5, and AS4 were classified as *A. stellatus* (PX091582, PX248706, PX248709, PX248713, and PX248716), while AN1 and AN2 were identified as *A. nidulans* (PX248708 and PX248715). In addition, AC1 (PX093629) and AO1 (PX363094) were identified as *A. cristatus* and *A. oryzae*, respectively.

**Figure 3:**
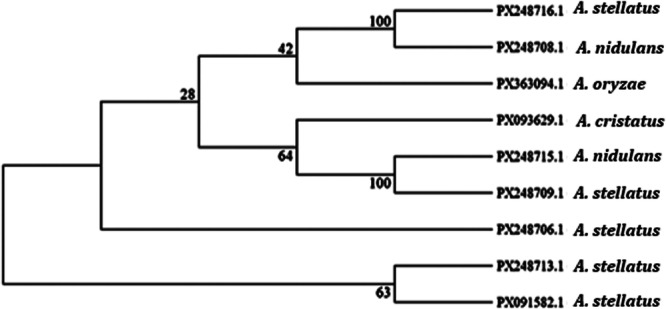
Phylogenetic tree generated using nucleotide sequence information of the ITS region of the conserved ribosomal DNA of *Aspergillus* isolates.

Subsequently, we developed a phylogenetic rooted tree using MEGA 12 software, employing the neighbor‐joining method based on sequence data from the amplified ITS region of nine isolated *Aspergillus* species. Additionally, we conducted an alignment with a consensus region through multiple sequence alignment using the CLUSTAL W program, which illustrated the evolutionary connections among the isolated strains (Figure 3). The robustness of the tree was evaluated using 1000 bootstrap replicates. Evolutionary distances for the neighbor‐joining tree were calculated using the maximum composite likelihood (MCL) model. Bootstrap values exceeding 60 at nodes confirmed the strong grouping of *A. stellatus* strains, while isolates such as *A. nidulans*, *A. oryzae*, and *A. cristatus* formed distinct, interconnected clusters with bootstrap values of 28, 42, and 64, respectively, indicating close evolutionary relationships and divergences within this section. Similarly, the maximum composite likelihood based on the nucleotide p‐distance of isolated *Aspergillus* strains demonstrated a high sequence similarity of *A. nidulans* with other strains, while showing moderate similarity to *A. oryzae*. Subsequently, *A. stellatus* exhibited close genetic relatedness within the group, with a p‐distance value ranging from 0.68 to 0.74, indicating intra‐species homogeneity. However, *A. cristatus* displayed moderate sequence similarity to the *A. stellatus* isolates (Supporting Information S1: Figure S1).

### 3.1 Antifungal Susceptibility Profile

Table 3 displays the MIC/MEC outcomes for the antifungal susceptibility profiles of five *A. stellatus*, two *A. nidulans*, one *A. cristatus*, and one *A. oryzae* isolate. The MIC values differed according to the *Aspergillus* strain tested. For *A. stellatus* strains, AMB showed a range of 0.5–2 µg/mL, whereas azoles such as PCZ, ITZ, and CAS had a lower MIC of 0.03 µg/mL. A slightly elevated MIC of 2 µg/mL for AMB was observed against isolated *A. stellatus* strains. For the two *A. nidulans* strains, the MICs for AMB were 0.125 and 2 µg/mL, respectively, with ITZ and VOR showing 1 µg/mL MIC. In the case of the single *A. cristatus* isolate, the MIC for AMB, ITZ, and VOR was 0.5 µg/mL; for RUV, it was 0.0625 µg/mL; for PCZ, it was 0.25 µg/mL; and the MEC for CAS was 0.031 µg/mL. Meanwhile, for the single *A. oryzae* isolate, the MIC for RUV was 0.03 µg/mL, for ITZ, VOR, and PCZ it was 0.125 µg/mL, the MEC for CAS was 0.062 µg/mL, and the MIC for AMB was 1 µg/mL.

**Table 3:** Antifungal susceptibility profile of tested antifungals against the nine isolated *Aspergillus* strains.

	MIC (µg/mL)										MEC (µg/ml)	
Isolated strains	AMB		ITZ		VOR		RUV		PCZ		CAS	
*A. stellatus* (*n* = 5)	Median	Range	Median	Range	Median	Range	Median	Range	Median	Range	Median	Range
	1	0.5–2	0.125	0.03–0.5	0.5	0.06–1	0.125	0.03–1	0.125	0.03–0.25	0.06	0.03–0.125
*A. nidulans* (*n* = 2)	Mean	Range	Mean	Range	Mean	Range	Mean	Range	Mean	Range	Mean	Range
	1.06	0.125–2	1	1	1	1	0.08	0.06–0.125	0.08	0.06–0.125	0.045	0.03–0.06
*A. cristatus* (*n* = 1)	0.5		0.5		0.5		0.0625		0.25		0.031	
*A. oryzae* (*n* = 1)	1		0.125		0.125		0.03		0.125		0.06	
*C. parapsilosis (ATCC 22019) (QC)*	0.5		0.125		0.03		0.06		0.06		0.25	
*C. krusei (ATCC 6258) (QC)*	2		0.25		0.125		0.25		0.5		0.125	

Abbreviations: AMB, amphotericin B; CAS, caspofungin; ITZ, itraconazole; MEC, minimum effective concentration; MIC, minimum inhibitory concentration; PCZ, posaconazole; QC, quality control; RUV, ravuconazole; VOR, voriconazole.

## 4 Discussion

Aspergillosis is a fungal infection caused by molds belonging to the *Aspergillus* genus, which takes advantage of weakened immune systems and presents a broad range of clinical symptoms. *A. nidulans* is an environmental saprotroph, if spores are inhaled, they can cause chronic and invasive pulmonary aspergillosis, especially in individuals with weakened immune systems, such as those with chronic granulomatous disease. In patients with prolonged immunosuppression, it spreads faster than other species (Pinheiro et al. 2023). The infection presents with persistent fever, chest pain, difficulty breathing, and coughing up blood, resembling other infectious diseases such as pulmonary tuberculosis. Other *Aspergillus* species, such as *fumigatus, flavus*, and *terreus*, cause similar symptoms in pulmonary aspergillosis. Respiratory aspergillosis is typically identified by demonstrating characteristic hyphae through direct microscopy using a KOH wet mount, along with macroscopic and microscopic features or molecular tools (Sedik et al. 2024). Identifying *Aspergillus* species requires examination of colony morphology and microscopic features, including vesicle shape, phialide arrangement, conidiophore structure, and conidial ornamentation. However, traditional methods have not always accurately identified all fungal species (Westblade et al. 2023). In this study, we phenotypically identified nine *A. nidulans* strains and reclassified them based on sequencing of the ribosomal ITS region, a universal DNA barcode marker for fungi. Phenotypic characteristics are subjective and influenced by environmental factors, such as media composition and incubation conditions (Balajee and Marr 2006). Modern diagnostic techniques, such as multilocus DNA sequence analysis and Matrix‐Assisted Laser Desorption/Ionization time‐of‐flight (MALDI‐TOF), can enhance traditional morphological identification to improve species identification accuracy (Geiser et al. 2007). The internal transcribed spacer is the most reliable region for identifying *A. nidulans* using species‐specific nucleotide polymorphisms. Hinrikson et al. (2005) found that the ITS region offers more precise species‐level identification than other genetic markers, with sequence identities ranging from 57.4%–98.1%. Al‐Ameri successfully identified *A. nidulans* strains by targeting 18S rRNA sequencing with ITS1 and ITS2 primers, resulting in a 600 bp DNA fragment that matched 100% with a standard isolate (Al‐Ameri 2024). However, other multilocus molecular phylogenetic analyses, such as partial β‐tubulin (BenA), calmodulin (CaM), and RNA polymerase II second largest subunit (RPB2), are widely used to identify *Aspergillus* section Nidulantes (Chen et al. 2016).

In the current study, we noted significant interspecies variation among nine phenotypically identified strains of *A. nidulans*. Upon sequencing the ITS region, these strains were reclassified into five strains of *A. stellatus* (with over 99% identity), two strains of *A. nidulans* (showing 100% and 99.54% sequence identity), one strain of *A. cristatus* (99.2% identity), and one strain of *A. oryzae* (100% identity). These species cannot be distinguished phenotypically because of their similar morphological characteristics. Molecular diagnostics play a crucial role in identifying rare and cryptic species of *Aspergillus*. Additionally, phylogenetic analysis and maximum likelihood methods confirmed the close genetic relationships among the isolated strains (*A. stellatus, A. nidulans, A. cristatus*, and *A. oryzae*). We determined the MIC for commonly used antifungals agents against the isolated strains and found significant variation in MICs for each. Notably, for *A. stellatus* strains, the MIC for azoles and MEC for CAS were slightly lower than those for AMB. Tavakoli et al. 2020 reported a higher MIC for AMB against the *A. nidulans* complex. Furthermore, Garcia‐Hermoso et al. (2022) noted variable MICs for CAS and AMB against *Aspergillus* section Nidulantes, including *A. nidulans* and *A. quadrilineatus*. Therefore, accurate diagnosis of phenotypically identified *A. nidulans* strains is essential for effective treatment. Molecular identification tools can help ascertain the precise cause of infection and identify pathogenic species. However, the limitation of this study is the small number of strains, highlighting the need for research with a larger sample size. Additionally, further multilocus gene sequence analysis, particularly of RBP‐2, is necessary for comprehensive molecular characterization. Despite these limitations, this study provides valuable data on *A. nidulans* and other phenotypically similar species from North India.

## 5 Conclusions

This study underscores the significance of sequencing the ITS1‐5.8sRNA‐ITS2 region to reveal significant differences between the phenotypic and molecular identification of rare *Aspergillus* isolates. Through sequencing, five *A. stellatus* isolates, two *A. nidulans* isolates, one *A. cristatus* isolate, and one *A. oryzae* isolate were accurately identified, which had been previously misclassified based on their phenotypic characteristics. These findings also highlight the limitations of phenotypic methods in distinguishing between closely related species. Moreover, the variations observed in the antifungal susceptibility profiles underscore the need for molecular‐based identification to ensure accurate clinical and epidemiological assessments, which can aid in appropriate antifungal susceptibility testing and treatment decisions. Additionally, incorporating molecular sequencing techniques into routine diagnostic procedures could greatly improve the precision of fungal identification, facilitating more targeted antifungal treatment. Future studies should aim to expand molecular databases for rare *Aspergillus* species to enhance identification accuracy and investigate the link between genetic variation and antifungal resistance patterns. Furthermore, the development of rapid, cost‐effective molecular diagnostic tools is crucial for timely clinical decision‐making and effective management of fungal infections.

## Author Contributions

**Aishwarya Nikhil:** investigation, and writing – original draft. **Atul Kumar Tiwari:** supervision, visualization, and data curation. **Pearl Parashar:** investigation and methodology. **Mohit Bhatia:** data curation, visualization, and supervision. **Ragini Tilak:** formal analysis and visualization. **Deepak Kumar:** formal analysis and visualization. **Sudhir Kumar Singh:** visualization, data curation, and supervision. **Roger J. Narayan:** writing – review and editing. **Munesh K. Gupta:** conceptualization, funding acquisition, data curation, writing – review and editing, supervision, visualization, and resources.

## Ethics Statement

The authors have nothing to report.

## Conflicts of Interest

The authors declare no conflicts of interest.

## Supporting information

Supporting File

## Data Availability

The data sets used and/or analyzed in the current study are available in the manuscript and supplementary files.
