# Supplementary material for: Molecular Characterization and Antifungal Profiling of Nine Phenotypic Aspergillus nidulans Isolates: A Case Series From North India
Source: Microbiologyopen. 2026 Jun 21;15(3):e70338. doi: 10.1002/mbo3.70338 (PMC13284283; doi:10.1002/mbo3.70338)
Supplement: Supplementary file 1 — Supporting File [file MBO3-15-e70338-s001.docx]

**Molecular Characterization and Antifungal Profiling of Nine Phenotypic *Aspergillus nidulans* Isolates: A Case Series** **from North India**

Aishwarya Nikhil^1^, Atul Kumar Tiwari^2^, Pearl Parashar^1^, Mohit Bhatia^3^, Ragini Tilak^1^, Deepak Kumar^4^, Sudhir Kumar Singh^4^, Roger J. Narayan^5*^, Munesh K. Gupta^1*^

^1^Mycology research group, Department of Microbiology, Institute of Medical Sciences, Banaras Hindu University, Varanasi, India-221005 [aishwaryanikhil1995@gmail.com](mailto:aishwaryanikhil1995@gmail.com); [pparashar1910@gmail.com](mailto:pparashar1910@gmail.com); [tilakragini28@gmail.com](mailto:tilakragini28@gmail.com); [muneshg.micro@bhu.ac.in](mailto:muneshg.micro@bhu.ac.in)

^2^School of Chemistry, Tel Aviv University, Tel Aviv-Yafo 6997801, Israel [atulkumartiwari.chembio@gmail.com](mailto:atulkumartiwari.chembio@gmail.com)

^3^Department of TB and Respiratory Medicine, Sir Sunderlal Hospital (BHU), Varanasi, India 221005. [mohitbhatia@bhu.ac.in](mailto:mohitbhatia@bhu.ac.in);

^4^Viral Research Diagnostic Laboratory, Department of Microbiology, Institute of Medical Science, Banaras Hindu University, Varanasi, India 221005. [deepak.micro@bhu.ac.in](mailto:deepak.micro@bhu.ac.in); [sudhirku@bhu.ac.in](mailto:sudhirku@bhu.ac.in).

^5^Joint Department of Biomedical Engineering, North Carolina State University, Raleigh, NC, USA [rjnaraya@ncsu.edu](mailto:rjnaraya@ncsu.edu)

**Corresponding Author:** [muneshg.micro@bhu.ac.in](mailto:muneshg.micro@bhu.ac.in) (Munesh Kumar Gupta), [rjnaraya@ncsu.edu](mailto:rjnaraya@ncsu.edu) (Roger J. Narayan)

**Supplementary File**

**Table S1.** List of *Aspergillus* isolates with their GenBank accession number and percentage identity.

| **S. No.** | **Isolate ID.** | **Isolation site** | **Morphological Identification** | **ITS Identification** | **Gene ID** | **% Identity** |
| --- | --- | --- | --- | --- | --- | --- |
| **1** | IMSMYCO**AS1** | BAL | *Aspergillus nidulans* | *Aspergillus stellatus* | PX091582 | 99.10% |
| **2** | IMSMYCO**AS2** | BAL | *Aspergillus nidulans* | *Aspergillus stellatus* | PX248706 | 99.13% |
| **3** | IMSMYCO**AS3** | BAL | *Aspergillus nidulans* | *Aspergillus stellatus* | PX248709 | 99.25% |
| **4** | IMSMYCO**AS4** | BAL | *Aspergillus nidulans* | *Aspergillus stellatus* | PX248716 | 99.53% |
| **5** | IMSMYCO**AS5** | BAL | *Aspergillus nidulans* | *Aspergillus stellatus* | PX248713 | 100% |
| **6** | IMSMYCO**AN1** | Sputum | *Aspergillus nidulans* | *Aspergillus nidulans* | PX248708 | 100% |
| **7** | IMSMYCO**AN2** | Sputum | *Aspergillus nidulans* | *Aspergillus nidulans* | PX248715 | 99.54% |
| **8** | IMSMYCO**AC1** | Sputum | *Aspergillus nidulans* | *Aspergillus cristatus* | PX093629 | 99.22% |
| **9** | IMSMYCO**AO1** | Sputum | *Aspergillus nidulans* | *Aspergillus oryzae* | PX363094 | 100% |


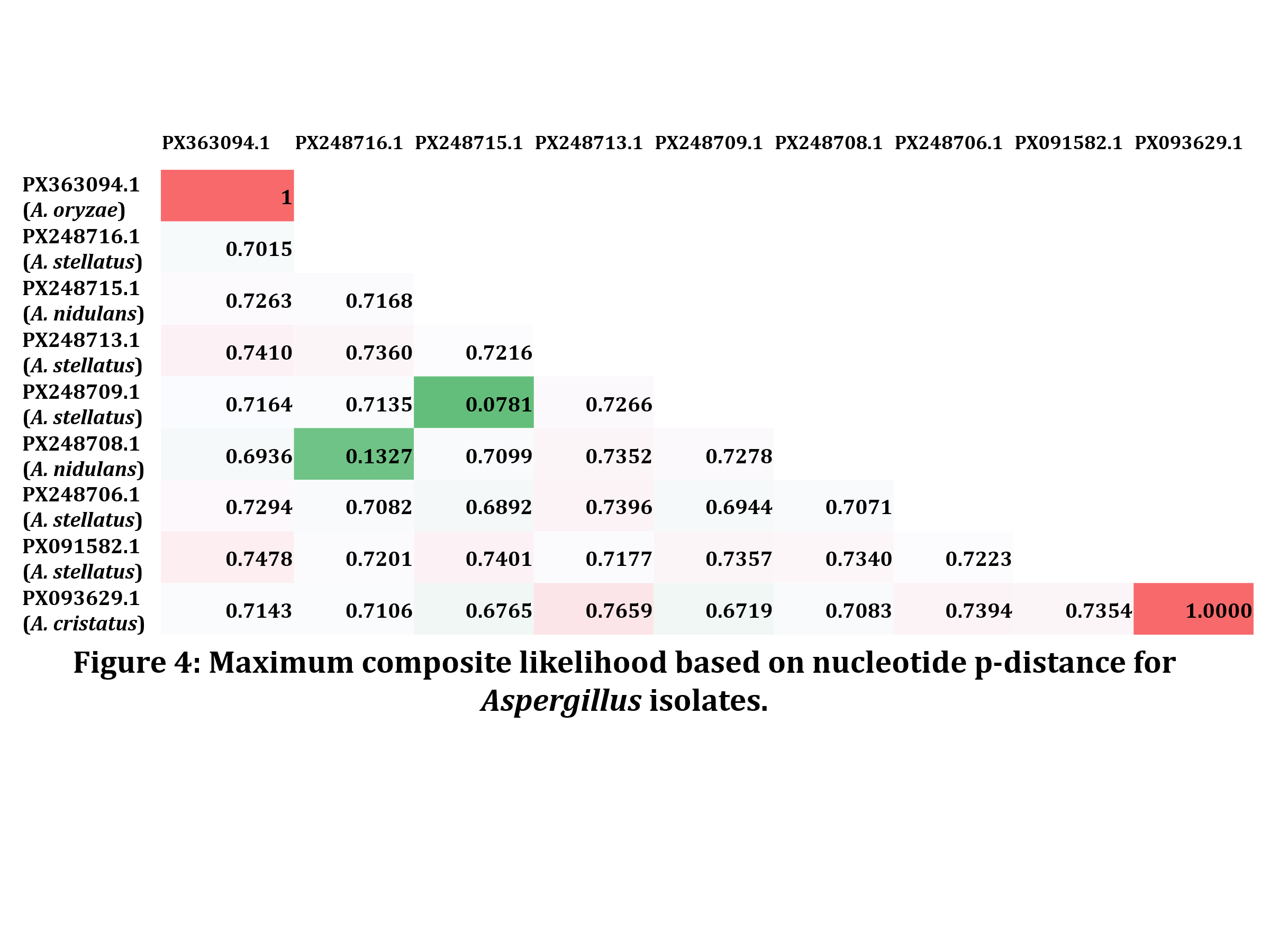


**Figure S1.** Maximum composite likelihood is based on nucleotide p-distance for *Aspergillus* isolates.
